# Trait choice profoundly affected the ecological conclusions drawn from functional diversity measures

**DOI:** 10.1038/s41598-017-03812-8

**Published:** 2017-06-16

**Authors:** Linhai Zhu, Bojie Fu, Huoxing Zhu, Cong Wang, Lei Jiao, Ji Zhou

**Affiliations:** State Key Laboratory of Urban and Regional Ecology, Research Center for Eco-Environmental Sciences, Chinese Academy of Sciences, Beijing, 100085 China

## Abstract

Although trait choice is crucial to quantify functional diversity appropriately, the quantitative methods for it are rarely compared and discussed. Meanwhile, very little is known about how trait choice affects ecological conclusions drawn from functional diversity measures. We presented the four methods of trait selection as alternatives to the ordination axis-based method, which directly identify a subset of key traits to represent the main variation of all the traits. To evaluate their performance, we compared the closeness of association obtained by different methods between species richness and functional diversity indices (FAD, FD, Q, FDis) in the six ecosystems. The evaluation was also benchmarked against the results obtained by calculating the possible indices using all the trait combinations (the complete search method). We found that the trait selection methods were potential alternatives to axis-based method to gain a mechanistic understanding of functional responses and effects of traits, while these methods as well as the axis-based method possibly use mismatched information to interpret the investigated ecosystem properties. Trait choice profoundly affected the ecological conclusions drawn from functional diversity measures. The complete search method should be used to assess the rationale of different trait choice methods and the quality of the calculated indices.

## Introduction

Functional diversity is increasingly recognized as an important approach for understanding the mechanisms of species coexistence or community assembly^1–3^, and characterizing the functional responses and effects of biological communities^4–8^. Appropriately quantifying functional diversity is often very difficult, especially when we have no clear knowledge about the functional links of organisms in specific ecosystems^9, 10^. Functional diversity is often represented by the value, range, and relative abundance of functional traits in a given community or ecosystem^5, 11^, when the ecological functions of organisms cannot be directly measured usually. Although trait selection will profoundly affect the value of functional diversity measures, this influence is rarely analyzed and discussed^10^.

Currently, three practices are often employed to select traits to measure functional diversity. First, traits considered to be important dimensions for plant ecological strategies are selected to measure functional diversity^12–15^. This method can facilitate the ecological synthesis across ecosystem, because these traits are usually measured in different ecosystems^12–15^. Second, all the measured traits are included into the functional diversity measures. This method often includes too many correlated or redundant traits, and will lead to losses of time, human and financial resources. Third, the ordination analysis (such as PCA, PCoA) is used to reduce the information of all the traits into independent axes^16, 17^. Then, the first several ordination axes are used as “traits” to measure functional diversity^16, 17^. This method is used especially when the number of traits is greater than the number of species, or qualitative traits are present in the trait matrix^16, 17^.

The recent researches suggest that some conceptual and methodological pitfalls are present with the ordination axis-based method^18^, which urge us to propose the four methods of choosing traits directly from a set of traits based on the Principal Component Analysis (PCA), and then these traits can be used to calculate functional diversity. Actually, Laliberté and Legendre^16^ suggest that if quantitative traits are available, these traits should be directly used to calculate functional diversity indices. However, this suggestion has not been well implemented. These trait selection methods will facilitate establishing the direct links between traits and ecosystem properties, enhance our understanding on the mechanistic links between traits and ecosystem properties^9^, and keep the trait number at the optimum one. As the potential alternatives to the axis-based method, these methods previously developed are underused to capture the main variations of all the traits.

Selecting a subset of traits to represent main variations of organisms’ functions requires certain criterion to measure its quality. The first three methods utilize RM coefficient, Yanai’s Generalized Coefficient of Determination (GCD) and RV coefficient separately to choose trait subsets that represent the total variation of all the traits^19–21^. The fourth method chooses the traits that had the highest loadings in the first several ordination axes^21^. These four methods constitute the trait selection methods. We compared the performance of these four methods with the axis-based method in linking functional diversity with ecosystem properties. We cannot expect that these four methods will identify the same trait combination. Therefore, we also assess the effect of the trait selection on the final ecological conclusion.

Petchey *et al*.^22^ propose that all the possible functional diversity indices should be calculated with every combination of traits, and then we can find the most suitable one that can be used to interpret ecosystem properties. This approach represents a pragmatic method to weight the traits by zero or one in all the trait combinations. Similarly, Maire *et al*.^17^ propose that all the possible functional spaces should be built with different numbers of ordination axes. We also think that this approach might assess the effects of trait choice on the ecological conclusions drawn from functional diversity^23^. Therefore, we calculated all the possible functional diversity indices using different numbers and identities of traits, and then we establish all the possible association between all these indices and ecosystem property. We call this method as the complete search. The comparison among the complete search, axis-based and trait selection methods would assess the effects of trait choice on the ecological conclusions.

We used species richness as the investigated ecosystem property. However, we are confident that the methodology and our conclusions can be easily transferred to other ecosystem properties. Understanding the relationship between species richness and functional diversity is important from different perspectives. First, manipulating species richness in controlled experiments will finally determine how communities perform the functions^24^, which needed to be quantified using trait diversity. Second, trait diversity, as an important representation of total resource use or functional diversity, needed to be quantified to understand how many species can coexist in specific ecosystems. The trait-based approach recently emerges to interpret variations of species richness along environmental gradients^3, 25^, which is based on the premise that favorable environments that allow wider ranges of traits or larger functional spaces will support more species. Therefore, species richness and functional diversity are conceptually interrelated. Third, quantifying the correlation between functional diversity and species richness is important to determine how much common information shared by them or how much they differed from each other, and to assure whether they are good proxies for each other^26, 27^. This quantification facilitates the understanding of their relative effects on ecosystem functioning, and forms a foundation for determining how much ecosystem functioning is provided by functional diversity beyond species diversity^26, 27^. This quantification is also pivotal to the implementation of biodiversity conservation, which need quantify the congruence between species richness and functional diversity^28, 29^. Finally, when a new functional diversity index is constructed, how it behaves with species richness is often tested. Most of previous studies use artificial data to explore the relationship between functional diversity and species richness^1, 16, 29, 30^. For most of ecosystems in nature, their real relationships and the effect of trait choice on them is rarely explored.

Here we first estimated the intrinsic dimensionality of plant traits in the six ecosystems, which was then used to determine the number of principal components (PCs) or key traits in the axis-based and trait selection methods. We used the trait selection methods to identify the key traits to represent the main variations of organisms’ functions. These key PCs and traits were used to calculate four functional diversity indices. We also calculated functional diversity indices using all the possible trait combinations. Finally, the closeness of associations between species richness and all the indices were determined to evaluate the performance of the proposed methods and assess the effects of trait choice on the ecological conclusions.

## Methods

### The data sets used

We used a total of six data sets^16, 31–37^. The detailed description for these data sets is in the Supplementary Information. The trait number of these data sets ranges from seven to thirteen. The traits in these data sets cover whole-plant, leaf, root, and regenerative traits (Supplementary Table S1). The ranges of species richness for the six data sets are summarized in the Supplementary Table S1. The abbreviations for all the traits are in the Supplementary Table S2.

### Trait correlations

The pair-wise Pearson correlations among traits were obtained using the psych package in R^38^. The percentage of significant correlations (P ≤ 0.05) in all the pair-wise relationships varied with the data sets. This percentage (62%) was highest in the Mount John data set (Supplementary Table S8), followed by the Jena data set (58%) (Supplementary Table S5). This percentage for the Rehoboth data set was 39% (Supplementary Table S6), which was higher than those of the Arizona data set (32%) (Supplementary Table S4) and the Lieu-dit Aravo data set (29%) (Supplementary Table S7). The lowest of this percentage was yielded in the Loess Plateau data set (26%) (Supplementary Table S3).

### The intrinsic dimensionality of traits

Cattell’s scree test^39^, Kaiser’s rule^40^, and Parallel analysis^41^ were used to estimate the intrinsic dimensionality of traits^42^. The analysis using the psych package in R^38^ revealed four dimensions for the traits in the five data sets. The exception was the Mount John data set where all the traits had only two dimensions (Supplementary Table S9). Correspondingly, four principal components (PCs) were used to quantify functional diversity for the former five data sets, and two PCs for the Mount John data set.

### Selecting trait subsets

The ordination axis-based method used the first four or two PCs to measure functional diversity (the PC method). If the original trait matrix can be successfully described by only *k* PCs, then it can often be represented by a subset of *k* traits, with a relatively small loss of information^21^. Therefore, the same numbers of traits were selected with an additional aim of keeping the consistency in the dimensionality of functional space.

As described in the Introduction, the four criterion used were the RM coefficient (the RM method), GCD (the GCD method), RV coefficient (the RV method), and the highest loadings in the first four or two PCs (the HL method).

In the PCA, the first *k* PCs are the *k* linear combinations of all the variables, which together maximize the percentage of total variation explained. The observations on all the variables then can be projected onto the subspace spanned by the *k* PCs. Similarly, the RM coefficient will choose an optimal subspace spanned by *k* traits, which maximize the percentage of total variation explained (equation 1)^20^.

1 $${\rm{RM}}=corr(X,{P}_{k}X)=\sqrt{\frac{tr({X}^{t}{P}_{k}X)}{tr({X}^{t}X)}}=\sqrt{\frac{\sum _{i=1}^{p}\lambda {({r}_{m})}_{i}^{2}}{\sum _{j=1}^{p}{\lambda }_{j}}}=\sqrt{\frac{tr({[{S}^{2}]}_{(\kappa )}{S}_{\kappa }^{-1})}{tr(S)}}$$

where *corr* denotes the matrix correlation;

*tr* is the matrix trace;

*X* is the original *n *×* p* matrix, and *p* is the number of original variables;

*P*_*k*_ is the matrix of orthogonal projections on the subspace spanned by a given *k*-variable subset;

${\rm{S}}=\frac{1}{n}{X}^{t}X$ is the *p *×* p* covariance (or correlation) matrix of the full data set;

*К* denotes the index set of the *k* variables in the variable subset;

S_*К*_ is the *k *×* k* principal submatrix of matrix S which results from retaining the rows/columns whose indices belong to *К*;

${[{S}^{2}]}_{(\kappa )}$ is the *k *×* k* principal submatrix of S^2^ obtained by retaining the rows/columns associated with set *К*;

λ_*i*_ stands for the *i*-th largest eigenvalue of the covariance (or correlation) matrix defined by *X*;

*r*_*m*_ stands for the multiple correlation between the *i*-th principal component of the full data set and the *k*-variable subset.

The GCD measures the closeness between the subspaces spanned by different trait combinations and the subspace spanned by *g* PCs^20^.

2 $${\rm{GCD}}=corr({P}_{k},{P}_{g})=\frac{tr({P}_{k},{P}_{g})}{\sqrt{k\cdot g}}=\frac{1}{\sqrt{kg}}\sum _{i\in G}{({r}_{m})}_{i}^{2}=tr({[{S}_{\{G\}}]}_{\kappa }{S}_{K}^{-1})/\sqrt{gk}$$

*P*_*g*_ is the matrix of orthogonal projections on the subspace spanned by *g* given principal components of the full data set.

*G* denotes the index set of the *g* principal components in the PC subset;

S_{G}_ is the *p *×* p* matrix of rank *g* that results from retaining only the *g* terms in the spectral decomposition of S that are associated with the PC indices in the set *G*;

[S_{G}_]_(*К*)_ is the *k *×* k* principal submatrix of S_{G}_ that results from retaining only the rows/columns whose indices are in *К*.

Considering two point configurations of all the observations, one is geometrically represented by the original trait matrix, another is defined by the matrix of *k* traits. The RV coefficient measures the similarity of the two point configurations to choose the optimum *k* traits, allowing for translations of the origin, rigid rotations and global changes of scale^19^. The RV coefficient can be defined as:

3 $${\rm{RV}}=corr(X{X}^{t},{P}_{k}X{X}^{t}{P}_{k})=\frac{1}{\sqrt{tr({S}^{2})}}\cdot \sqrt{tr{({[{S}^{2}]}_{(\kappa )}{[{S}_{\kappa }]}^{-1})}^{2}}$$

The subselect package in R was used to choose key traits based on RM coefficients, GCD and RV coefficient^43^. We performed the PCA to determine the traits having the highest loadings in the first two or four PCs using the vegan package in R^44^ (Supplementary Figure S1–S6).

Petchey *et al*.^22^ and Maire *et al*.^17^ advocate computing all the possible functional diversity indices using every trait combination or ordination axis. We followed them and calculated the possible functional diversity indices using all the trait combinations. We named this method as the complete search method (the CS method). Unsurprisingly, this method would obtain a best or equivalent performance compared with the axis-based and trait selection methods, and could serve as a benchmark method^23^. However, the CS method should be applied with great caution to avoid overemphasizing the mathematical relationships between functional diversity and ecosystem properties.

### Calculating functional diversity indices

We calculated four functional diversity indices (FAD, FD, Q, and FDis) for each quadrat or plot. The functional attribute diversity (FAD) is the sum of pair-wise standardized distances between species in attribute space^45^. FD is the total branch length of a functional dendrogram^46, 47^. The Rao’s quadratic entropy (Q) is the sum of pair-wise distances between species weighted by relative abundance^48, 49^. The functional dispersion (FDis) is the weighted mean distance of individual species to the weighted centroid of all species in multidimensional trait space, and weights here correspond to the relative abundances of the species^16^.

The average trait values of each species were used to calculate the functional diversity indices. The values of a trait were standardized to a mean of 0 and unit variance using z-transformation so that each trait had the same weight in functional diversity measures and the units of trait values had no influence^16^. We calculated the indices using the free software FDiversity^50^, the FD package in R^14^, and the R code provided by Petchey *et al*.^51^. The Euclidean distance and average linkage were chosen when a distance index and clustering algorithm were needed^51^.

### The performance of the proposed methods and the effect of trait choice on ecological conclusions

Different methods might identify different trait combinations to establish the associations between functional diversity and species richness. The performance of different methods was finally evaluated using the closeness of these associations. Therefore, the linear relationships were established between all the functional diversity indices and species richness for each data set. The coefficients of determination (COD or R^2^) in each linear regression were used to represent the closeness of their association. The variation in the closeness of these associations reflects the effect of trait choice. We used a two-way analysis of variance to assess the effect of trait choice on the closeness of associations between species richness and functional diversity, and whether or not this influence varies with functional diversity indices. We considered the six data sets as the replications for this analysis.

## Results

### The performance of the proposed methods

**Figure 1:**
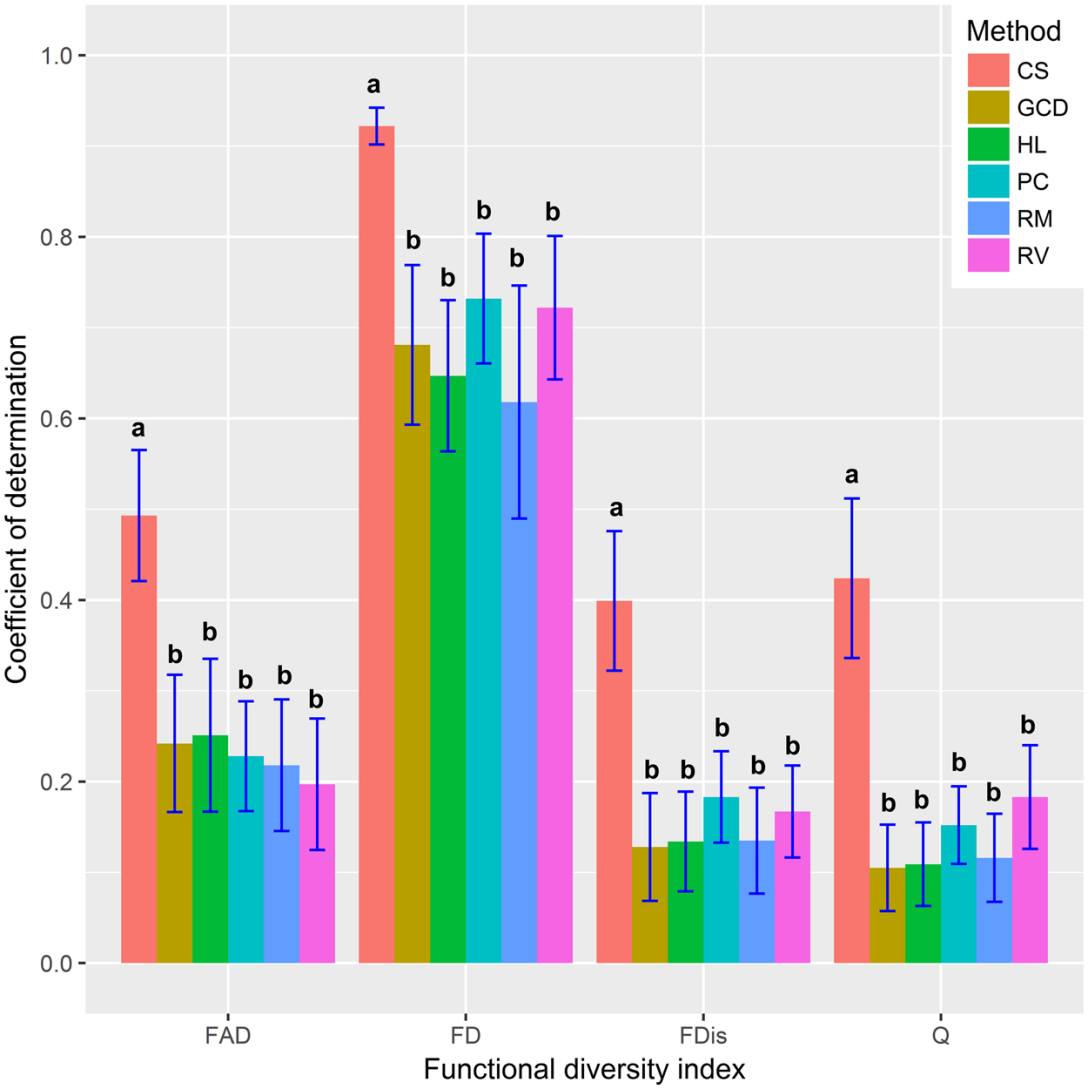
The average closeness of associations between species richness and functional diversity obtained by different methods across four functional diversity indices, FAD, FD, Q, and FDis and six ecosystems. For the CS method, the average was calculated using the highest closeness in each ecosystem. The blue lines indicate ± 1standard error. The different letters above the bars of one functional diversity indices indicate significant difference at the level of 0.05.

The first comparison can be made between the PC method and trait selection methods (Fig. 1). The analysis of variance suggests that no significant difference exist for the average closeness of association among these five methods. However, the average closeness of association obtained using these five methods is significantly lower than those obtained using the CS method. This result holds across different functional diversity indices.

### The effect of trait choice on ecological conclusions

**Figure 2:**
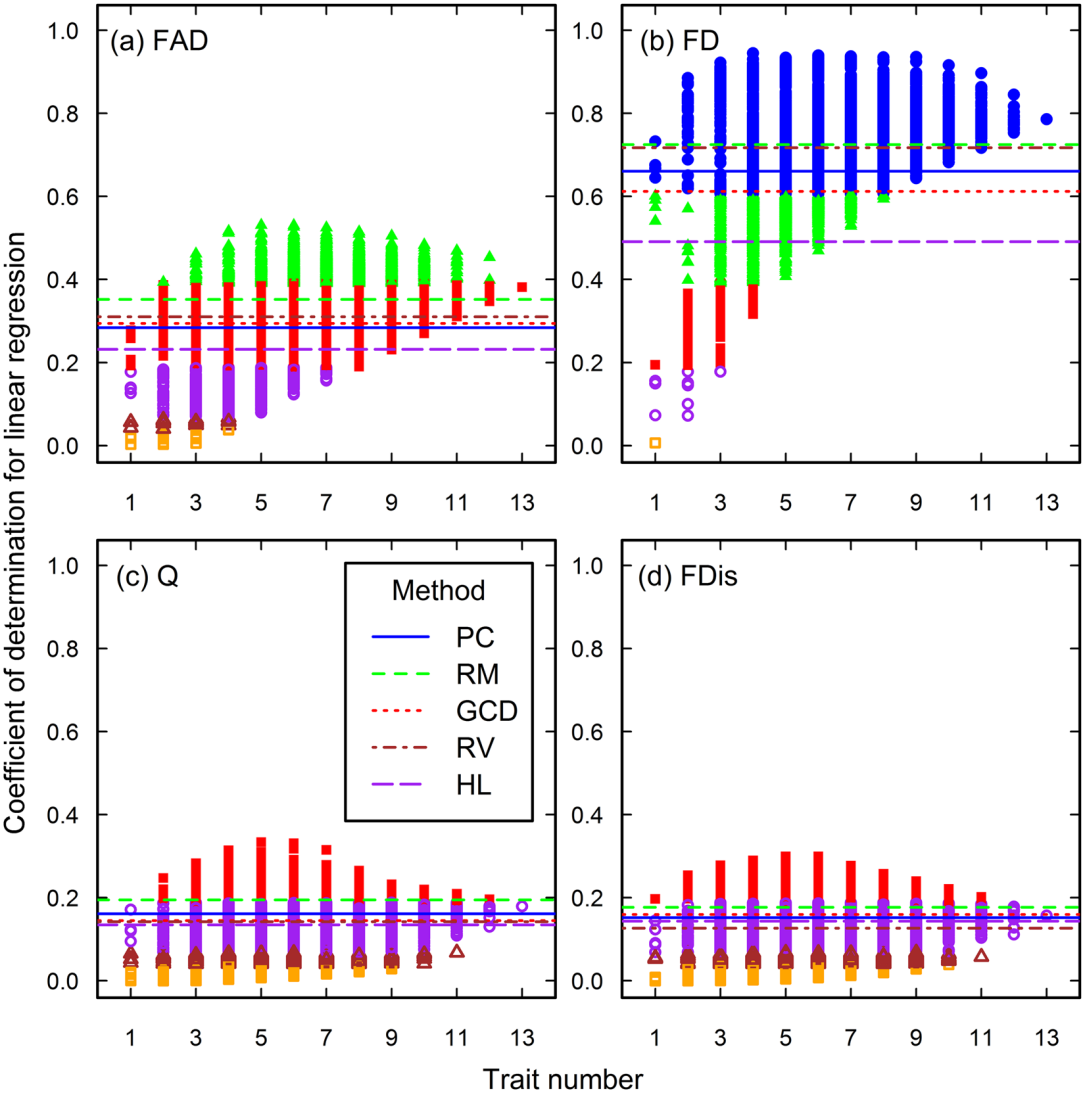
The closeness of associations between species richness and functional diversity in the Loess Plateau data set. The four functional diversity indices, FAD (**a**), FD (**b**), Q (**c**), and FDis (**d**), were calculated using different numbers and identities of traits. Each line in the plots donates the closeness of associations between species richness and one functional diversity index calculated using one subset of traits, which were determined using one of the methods, PC (principal components), RM (RM coefficient), GCD (Yanai’s Generalized Coefficient of Determination), RV (RV coefficient), and HL (the highest loads). One point in each plot represents the closeness of the association calculated using one trait combination in the analysis of the complete search. The points with different colors and symbol represent different levels of P values. Filled and blue circles, P ≤ 1.0 × 10^−20^; filled and green triangles, 1.0 × 10^−20^ < P ≤ 1.0 × 10^−11^; filled and red squares, 1.0 × 10^−11^ < P ≤ 1.0 × 10^−5^; open and purple circles, 1.0 × 10^−5^ < P ≤ 0.01; open and brown triangles, 0.01 < P ≤ 0.05; open and orange squares, P > 0.05.

**Figure 3:**
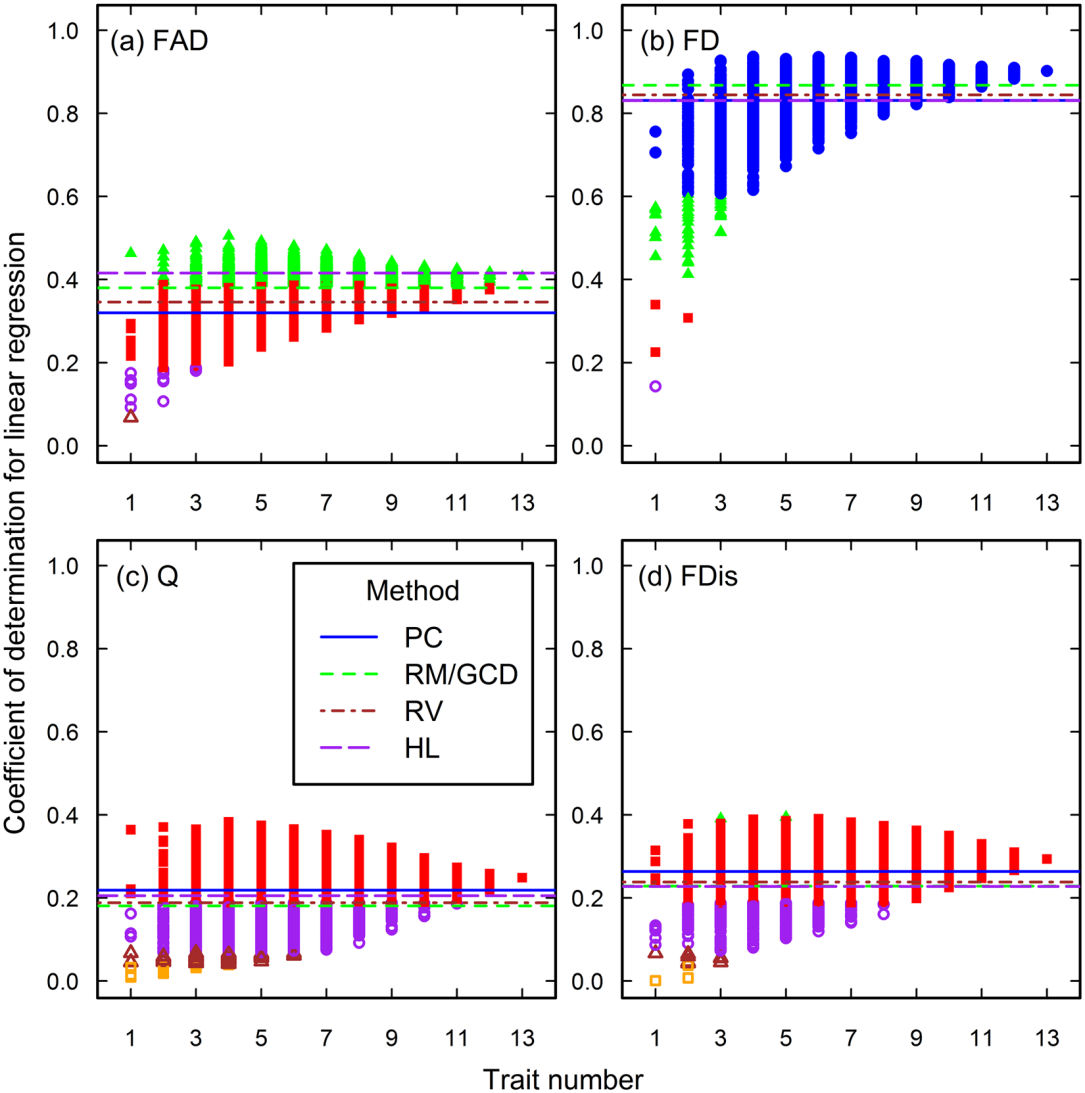
The closeness of associations between species richness and functional diversity in the Arizona data set. See the caption of Fig. 1 for the meanings of each plot, line and point.

**Figure 4:**
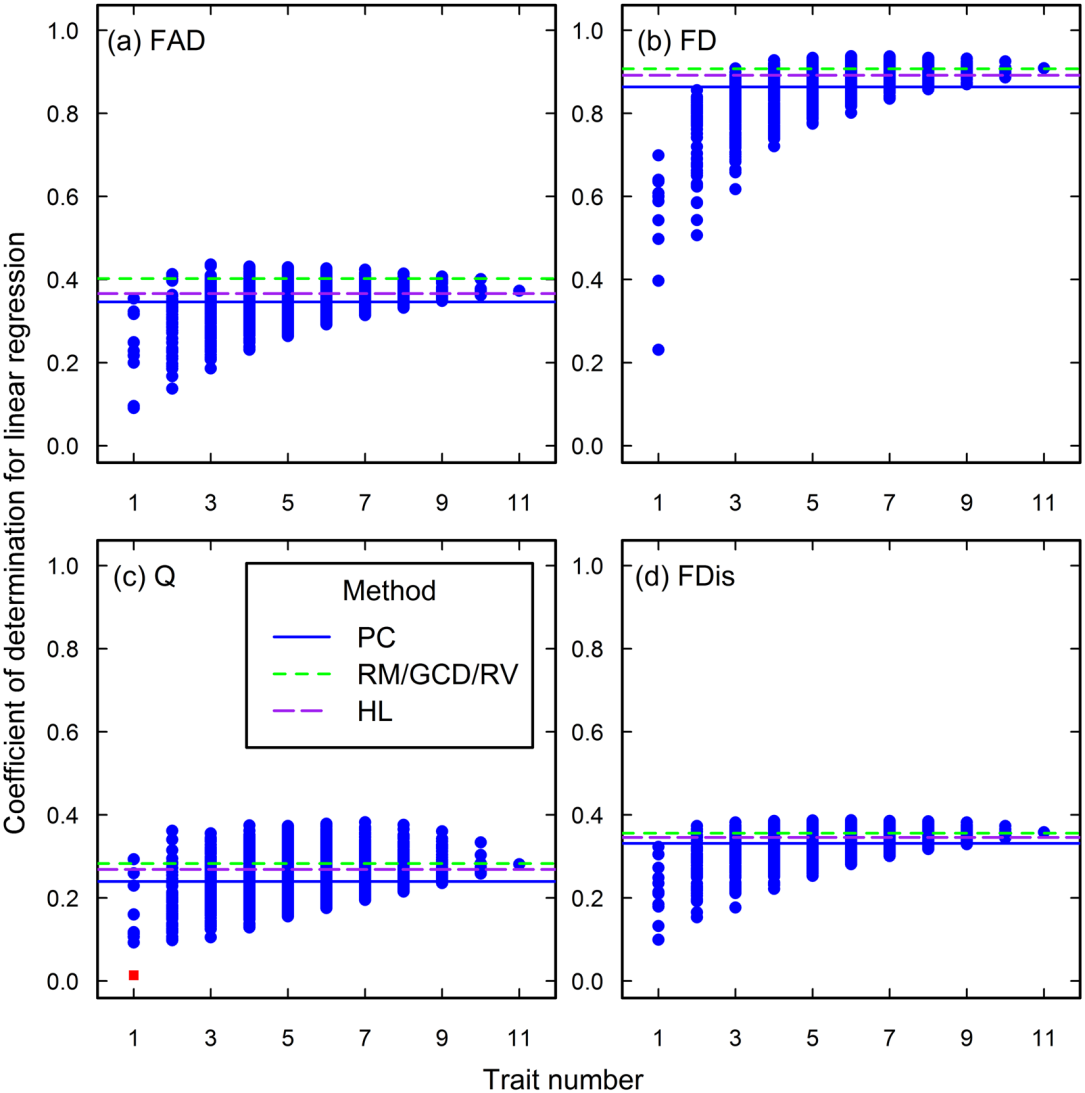
The closeness of associations between species richness and functional diversity in the Jena data set. See the caption of Fig. 1 for the meanings of each plot, line and point.

**Figure 5:**
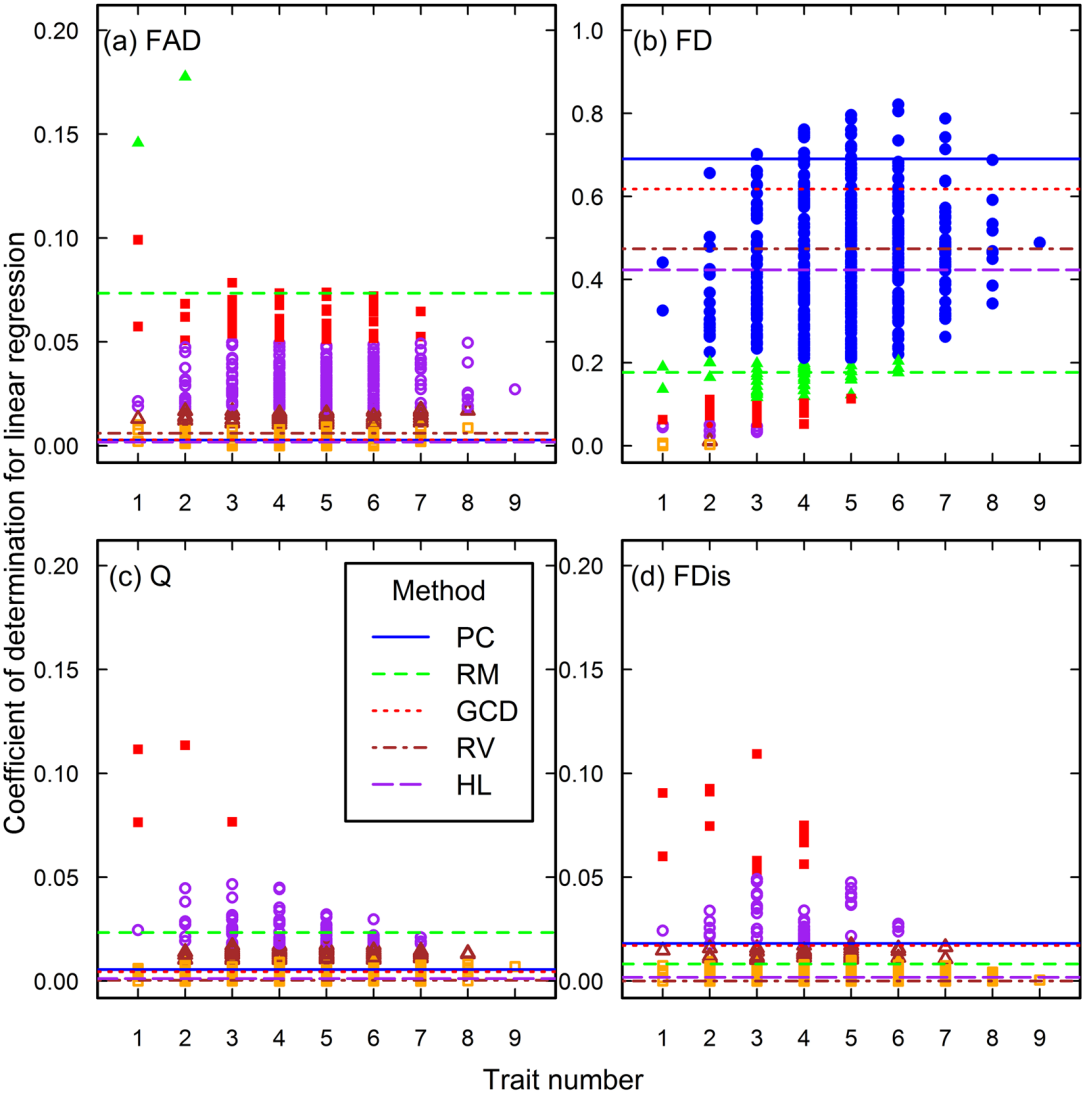
The closeness of associations between species richness and functional diversity in the Rehoboth data set. See the caption of Fig. 1 for the meanings of each plot, line and point. Note the ranges of coefficients of determination in the plot a, c, and d, which are different from that of the plot b.

**Figure 6:**
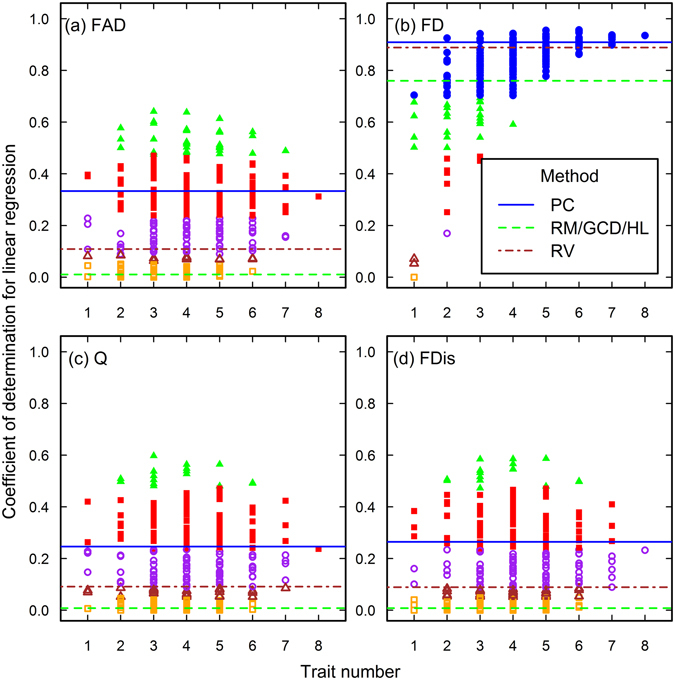
The closeness of associations between species richness and functional diversity in the Lieu-dit Aravo data set. See the caption of Fig. 1 for the meanings of each plot, line and point.

**Figure 7:**
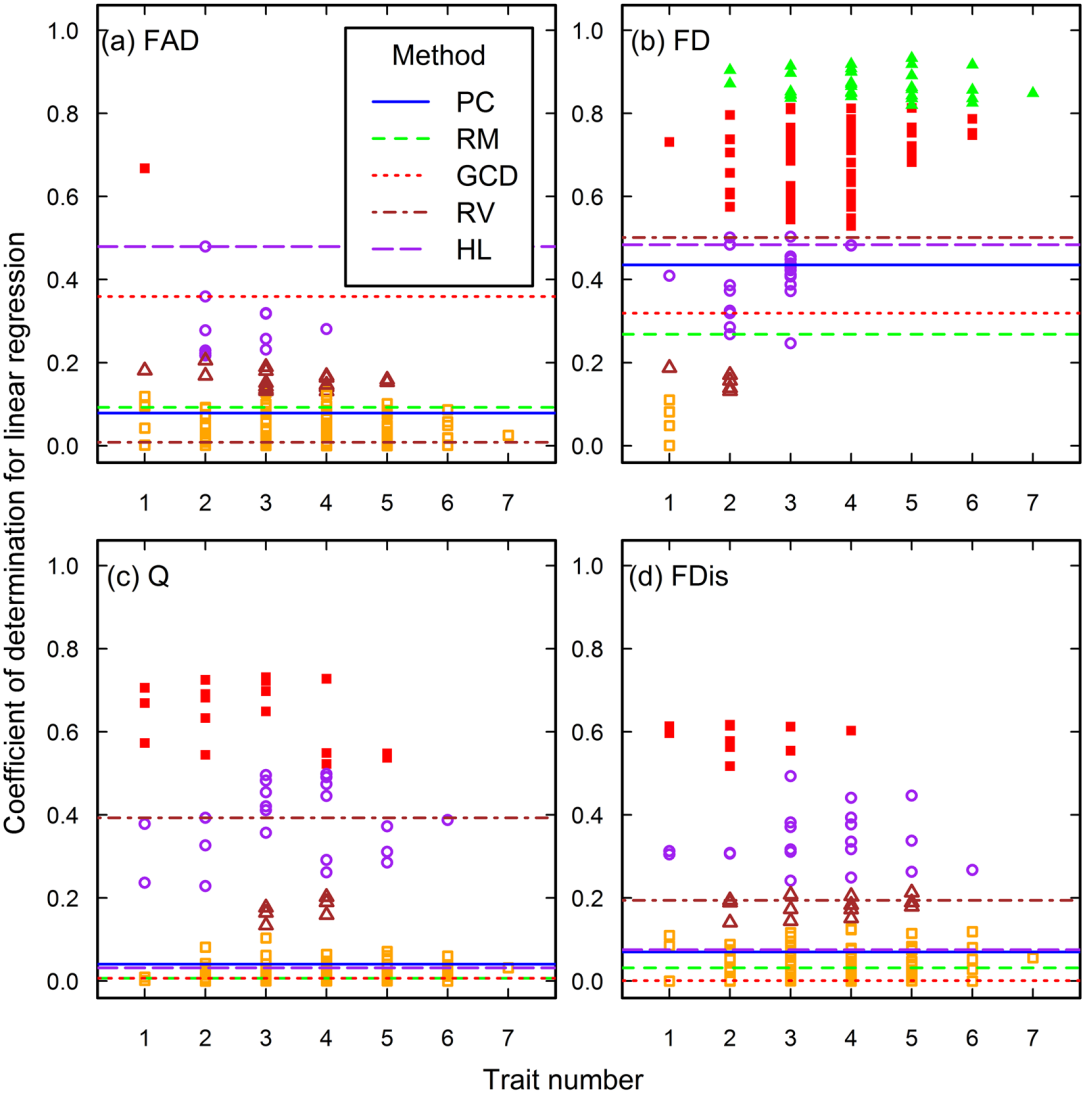
The closeness of associations between species richness and functional diversity in the Mount John data set. See the caption of Fig. 1 for the meanings of each plot, line and point.

The closeness of associations between functional diversity and species richness vary with the trait combinations (Figs 2–7). As the trait number increases, the association closeness increase and their variations decrease for the Loess Plateau (Fig. 2), Arizona (Fig. 3) and Jena datasets (Fig. 4), and FD in other three datasets (Figs 5b, 6b and 7b). For the FAD, Q and FDis in the Rehoboth (Fig. 5a, c, d) and Mount John dataset (Figs 7a, c, d), closer associations can be obtained by using the combinations of fewer traits.

### The best trait combination

The trait combinations producing the association of the highest closeness were totally identified by the complete search and completely different from those of the four trait selection methods (Tables 1 and 2). The best combinations were also mostly different for the four functional diversity indices (Table 2). Only the FAD and Q shared the same combinations in the Rehoboth data set and the Lieu-dit Aravo data set. However, some traits existed in all the best combinations of the same data sets, i.e. the Hv in the Loess Pleateau data set, the L15N in the Arizona data set, the RDepth, SMF and SLA in the Jena data set, the ACD and SLA in the Rehoboth data set, the Hv, LAngle, and LN in the Lieu-dit Aravo data set.

**Table 1:** The subsets of traits determined by the ordination axis-based and trait selection methods. See the abbreviations for traits in the Table S2 in the Supplementary Information.

Data sets	Methods	Traits identified	Percentage of total variation explained (%)^1^
Loess Plateau, China	PC	Four principal components	72.1
	RM	Hv, Area, LP, RLateral	61.4
	GCD	Area, LDMC, Rdepth, RMF	63.0
	RV	Area, LDMC, LN, Rlateral	71.7
	HL	LDMC, Rdepth, Area^2^	51.0^3^, 52.3^3^, 60.1^3^
Arizona, USA	PC	Four principal components	61.3
	RM	Hv, SRL, LN, L15N	50.6
	GCD	Hv, SRL, LN, L15N	51.6
	RV	Hv, LDMC, SRL, LN	58.3
	HL	LN, SRL, Hv, FlrDuration	49.3, 43.1, 56.1
Jena, German	PC	Four principal components	80.3
	RM	RDepth, LMF, SLA, LNa	73.9
	GCD	RDepth, LMF, SLA, LNa	75.1
	RV	RDepth, LMF, SLA, LNa	83.4
	HL	LMF, LNa, RDepth, SeedEmerg	71.9, 66.1, 81.7
Rehoboth, Namibia	PC	Four principal components	69
	RM	Height, LWRatio, Area, DiaLen	57.3
	GCD	Height, LWRatio, LT, SLA	52.4
	RV	ACD, LT, SpiLen, SeedLen	62.5
	HL	SeedLen, Area, LWRatio, SLA	56.0, 42.4, 57.1
Lieu-dit Aravo, France	PC	Four principal components	75.7
	RM	Hv, Spread, Area, SLA	66.2
	GCD	Hv, Spread, Area, SLA	63.6
	RV	Spread, LAngle, SLA, SeedMass	69.1
	HL	Hv, Spread, Area, SLA	66.2, 63.6, 68.7
Mount John, New Zealand	PC	Two principal components	74
	RM	LS, Area	60.2
	GCD	Hr, LS	45.4
	RV	LN, LP	74.4
	HL	LN, Hr	59.7, 37.3, 62.2

^1^For the methods of PC, RM and RV, the percentage indicates the explained information of original trait matrix. For the GCD method, the percentage indicates the explained information of reserved principal components.

^2^In the Loess Plateau data set, area of a leaf (Area) had the highest loadings in the third and fourth principal components (Figure S1).

^3^The three numbers in the HL row were quantified using the RM coefficients, GCD and RV coefficients.

**Table 2:** The best subsets of traits determined by the complete search method. Functional diversity indices calculated using these traits had the association of the highest closeness with species richness. See the abbreviations for traits in the Table S2 in the Supplementary Information.

Data sets	Functional diversity indices	Traits identified	Trait Number
Loess Plateau, China	FAD	Hv, Area, LDMC, LP, RMF	5
	FD	Hv, LDMC, LC, LFp	4
	Q	Hv, Area, SLA, LC, LFp	5
	FDis	Hv, Area, SLA, LC, RMF	5
Arizona, USA	FAD	Hv, FlrDuration, L15N, RN	4
	FD	Hv, LDMC, LN, L15N	4
	Q	Area, FlrDuration, L13C, L15N	4
	FDis	Area, LDMC, FlrDuration, L13C, L15N	5
Jena, German	FAD	RDepth, SMF, SLA	3
	FD	RDepth, RMF, ShootMass, SMF, SLA, LN	6
	Q	RDepth, RMF, ShootMass, SMF, LMF, SLA, LN	7
	FDis	RDepth, ShootMass, SMF, LMF, SLA, LN	6
Rehoboth, Namibia	FAD	ACD, SLA	2
	FD	height, ACD, LWRatio, LT, SLA, SeedLen	6
	Q	ACD, SLA	2
	FDis	ACD, LWRatio, SLA	3
Lieu-dit Aravo, France	FAD	Hv, LAngle, LN	3
	FD	Hv, LAngle, LT, SLA, LN, SeedMass	6
	Q	Hv, LAngle, LN	3
	FDis	Hv, LAngle, LT, LN, SeedMass	5
Mount John, New Zealand	FAD	Hr	1
	FD	Hr, LDMC, LN, LP, SLA	5
	Q	LDMC, LP, SLA	3
	FDis	LP, SLA	2

The analyses of the trait combinations of the five datasets having four trait dimensionalities in the Tables 1 and 2 revealed that the selected traits are from at least three independent dimensions in most cases, while some of these traits are also correlated (Supplementary Tables S3–S8), for example, specific leaf area (SLA) and leaf nitrogen concentration (LN) in different datasets.

SLA is included in all the datasets (Supplementary Table S2). Height (either vegetative or reproductive) and leaf area are included in the five datasets except the Jena dataset. Seed mass is included in the four datasets except the Loess Plateau and Rehoboth datasets. These four traits are considered as the key ones that can be used to represent the leading dimensions of plant ecological strategies. However, these four traits are not always present in the best trait combinations (Table 2).

## Discussion

For selecting traits to associate functional diversity with species richness, the trait selection methods are comparable to the ordination axis-based method. When compared with the complete search method, the performance of these five methods is poorer. The closeness of associations between functional diversity and species richness vary largely with the trait combinations, showing that trait choice profoundly affects the ecological conclusions drawn from functional diversity. The combinations of fewer traits are likely to better interpret the variations of species richness, implying that trait identity might be more important than trait number. The selected traits can be from different trait dimensions, while some of which can be also correlated. The traits previously considered as the key ones to represent the leading dimensions of plant ecological strategies are not always present in the best trait combinations, suggesting the traits functions differently in different contexts.

### The performance of different methods

Our results suggest that the trait selection methods are potential alternatives to ordination-axis based method. These five methods would similarly try to find the best solutions to represent the variation of all the traits^19–21^. While the trait selection methods directly identify traits, the ordination axis-based method produced the complexes of traits as ordination axes. Therefore, the trait selection methods can facilitate the establishment of the mechanistic links between traits and ecosystem properties^9^. However, the trait selection methods are presently limited to the quantitative traits because of the data requirement of PCA, whereas the ordination axis-based method can dealing with different types of traits via the Principal Coordinate Analysis (PCoA)^16, 17^. To widen the application of the trait selection methods, they should be extended to other types of traits.

The results of the CS method present the potential space where the axis-based and trait selection method can be improved. When the axis-based and trait selection methods determined the key traits or ordination axes that capture the main variations of plant functions, the functional links of these traits or axes with investigated ecosystem properties are usually not guaranteed in common practices of calculating functional diversity indices as in this study. All the biotic and abiotic factors shape the traits, and thus trait dimensionality in an ecosystem^18^. However, only one to several ecosystem properties are usually investigated, which probably only some of the traits are strongly associated with these ecosystem properties. We think that there exist a mismatch between the key information that axis-based and trait selection methods extract to represent the variation of all the traits and the whole information required to interpret one ecosystem property. This mismatch can be validated by the inconsistency of trait combinations between the CS method and these five methods (Tables 1 and 2). These five methods would possibly include the information not needed and discard the information needed to interpret ecosystem properties. While it is important to show that functional diversity respond to or affect ecosystem properties, it is pivotal to identify the traits that have clear links with ecosystem properties to gain a mechanistic understanding of functional response and effect of organisms^9, 10, 51^. Our results show the limitations of statistical methods for establishing these mechanistic links, which allow statistical criterion to make a decision on trait choice. We think that only by experimental methods we could establish the mechanistic links of traits with ecosystem properties, while these statistical methods are still of great value considering the ubiquity of observation studies. However, we must acknowledge the limitations of statistical methods, before we can truly understand functional responses and effects of organisms and biological communities.

### The effect of trait choice

The results of the CS method show that trait choice profoundly affects the ecological conclusion drawn from functional diversity. As advised by Petchey *et al*.^22^ and Maire *et al*.^17^, we advocated computing all the possible functional diversity indices using every trait combination or ordination axis to assure the suitability and rationale of the trait selection and ordination axis-based method^23^. We think that if an appropriate original trait matrix is constructed, the closeness of association will increases and their variation will decrease between functional diversity and investigated ecosystem properties when more traits are included into the functional diversity indices. We can see that only in the six analyses (Figs 3a, b, 4a, b, d, 6b), the association closeness gained by axis-based and trait selection methods are similar to the highest closeness obtained by the CS method. The CODs produced by the CS method for these six analyses showed similar patterns. Specifically, the CODs in the large trait numbers in these analyses converged better and were closer to that of the best combination in the same analysis. In other words, if the CODs diverged more in the larger trait numbers, the possibility of constructing an inappropriate trait matrix would increase.

Increasing trait number did not necessarily produce association of higher closeness between species richness and functional diversity. A high incidence existed that the low dimensional function diversity indices surpassed those high dimensional ones. Several ecologists also find that single-trait functional diversity indices can outperform multiple-trait functional diversity indices in explaining the variations of ecosystem properties^22, 53, 54^. These results implied that trait identity might be more important than trait number to measure functional diversity.

### The context dependency of ecological functions of traits

As the dominant factors shaping the traits and trait dimensionality are usually different in different contexts^18^, we cannot expect to use the same traits to represent the main variation of plant functions and interpret the investigated ecosystem properties across different ecosystems. We find that the traits (such as height, leaf area, specific leaf area and seed mass) previously considered to be important dimensions for plant ecological strategies are not always present in the best trait combinations that capture the main variations of all the traits or best interpret the variation of species richness (Tables 1 and 2). Therefore, the understanding for the dimensionality and functional links of traits should be further advanced, especially in local ecosystems. The traits truly important to interpret the investigated ecosystem properties can be identified only by the exploration in the local ecosystems, not by borrowing the results from other studies or global analyses.

The six ecosystems in this study differ from each other in the main limiting resources. Therefore, the main traits taking an important role in constructing the functional spaces also vary with these ecosystems. For the Loess Plateau of China, soil erosion, water and nutrient deficit are the main limiting factors of plant^55–57^. Root distribution, leaf economics spectrum, height and leaf area are important traits of extant species. Water and nitrogen are the primary limiting resources in the ecosystem of Arizona, USA^35, 36^. The traits associated with the water and nutrient absorption and usage (e.g. SRL, L15N and L13C) are important to determine the specie performance. The Jena experiment was established by growing plants of different biodiversity gradient^33, 34^. Therefore, the competitive advantage for light and space and the rapid growth are important for species’ establishment, which implying the important roles of root distribution, shoot mass and leaf economics spectrum. The snowmelt, growing-season-length, zoogenic and physical disturbance shape the plant community in the ecosystem of Lieu-dit Aravo, France^32^. Therefore, the height, leaf area, angle and economics spectrum are important to determine the species’ existence. Although the plant communities in the ecosystems of Rehoboth, Namibia^37^ and Mount John, New Zealand^16^ are shaped with the similar limiting factors, grazing intensity and soil resources, the traits (such as SLA and height) do not play similar roles in determining the species’ occurrence in these two ecosystems. This result can be explained by the spatial-temporal variations and complexity^37, 58–60^.

### The independency of ordination axes

We found that in most cases, the selected traits are from at least three independent dimensions, while the trait correlations were widely present. These results accord with the whole-plant perspective, which proposes that plant coordinates different functional dimensions to uses the resources effectively and cope with all the limiting factors^18^. Although the traits function differently, they can be correlated from the whole-plant perspective^18, 52^. By an analysis of the most comprehensive species-trait matrix to date, Díaz *et al*.^61^ also find that the stem density, leaf area and diaspore size correlate with the plant height and leaf economics spectrum, although these three traits are previously considered as the independent trait dimensions. These results urge us to reconsider the usage of independent axes in the calculation of functional diversity indices. However, traits from different functional dimensions should be measure to improve the quality of functional diversity indices.

## Conclusions

While the trait selection methods are alternatives to the ordination axis-based method to gain a mechanistic understanding of functional links of traits, these methods as well as axis-based methods possibly uses the mismatched information to interpret the investigated ecosystem properties. As the trait choice profoundly affects the ecological conclusion drawn from functional diversity, we propose that the complete search method should be used to assess the quality of calculated functional diversity indices. We think that we can truly understand functional response and effect of traits from a mechanistic perspective only through experimental methods, while the trait selection methods is still of great value because the observation researches are ubiquitous. We expect that we can obtain a deeper understanding of functional response and effect of traits by replacing ordination axes with traits to calculate functional diversity indices.

## Electronic supplementary material

Supplementary Information
