# Supplementary material for: Trait choice profoundly affected the ecological conclusions drawn from functional diversity measures
Source: Sci Rep. 2017 Jun 16;7:3643. doi: 10.1038/s41598-017-03812-8 (PMC5473860; doi:10.1038/s41598-017-03812-8)
Supplement: Supplementary file 1 — Supplementary Information [file 41598_2017_3812_MOESM1_ESM.pdf]

Supplementary Information

Trait choice profoundly affected the ecological conclusions drawn from functional diversity measures

Linhai Zhu, Bojie Fu *, Huoxing Zhu, Cong Wang, Lei Jiao, Ji Zhou

* Corresponding author

Bojie Fu

Email address: bfu@rcees.ac.cn

Telephone: +86-10-62923557

The description of the data sets used

The Loess Plateau data set was collected in semi-arid grasslands of the Yangjuangou catchment (36°42′ N, 109°31′ E) in the middle of the Loess Plateau of China. In August and September of 2013 and 2014, during the peak of vegetative growth, 96 quadrats (1 m × 1 m) were surveyed to determine species composition and abundance. In each quadrat, we identified as many species as possible and visually estimated their abundance (vertical projective coverage). For the 28 species present in the quadrat, we measured vegetative height, root mass fraction, area of a leaf, leaf dry matter content, specific leaf area, leaf nitrogen concentration, leaf carbon concentration, leaf phosphorous concentration, force to tear of leaves, force to punch of leaves, force to tear of roots, depth of roots, lateral extent of roots (Pérez-Harguindeguy et al. 2013).

The Arizona data set was sampled in the herbaceous understory of ponderosa pine forests in the Coconino National Forest, Arizona, USA (Shipley et al. 2011). We used the 13 quantative traits and abundances of 79 species in 96 quadrats. The percentage cover of species was visually estimated to obtain their relative abundances. The traits are vegetative height, area of a leaf, leaf dry matter content, specific leaf area, specific root length, seed mass, flowering duration, leaf carbon concentration, leaf nitrogen concentration, fine-root carbon concentration, fine-root nitrogen concentration, leaf $\delta^{13}C$, and leaf $\delta^{15}N$.

The Jena Experiment is one of the longest running biodiversity experiments in Europe (Roscher et al. 2004; Weigelt et al. 2010). The experimental site is located on the floodplain of the Saale river (altitude 130 m NN) at the northern edge of Jena (Jena-Löbstedt, Thuringia, Germany). In this experiment, a gradient of plant species richness (1, 2, 4, 8, 16 and 60 species) and functional richness (1, 2, 3, 4 functioinal groups) was estabalished in the 82 grassland plots of 20 × 20 m in May 2002. The biomass was harvested and sorted to species from 3 - 4 subplots of 0.2 × 0.5 m per experimental plot from September 2002 to August 2008. The harvest was impelemented once in 2002 and twice in the other years (once in May or June, once in

August). The original biomass data includes a total of 3960 observations. Deleting the observations with missing values yielded the abundance data of 3281 observations. The original data set contains the information on 60 species common to Central Europe semi-natural grasslands. Only the trait values of 58 species were extracted from the literature (Heisse et al. 2007). Therefore, we anylysed this data set using the 11 traits and abundances of 58 species. The triats are seed mass, time to seedling emergence, depth of roots, root mass fraction (converted from shoot-root ratio), dry mass per shoot, stem mass fraction (stem dry weight per shoot dry weight), leaf mass fraction (leaf dry weight per shoot dry weight), specific leaf area, leaf area ratio (the product of specific leaf area and leaf mass fraction), leaf nitrogen contentration per leaf dry weight, leaf nitrogen contentration per leaf area.

The Rehoboth data set was recorded in semi-aird rangelands near the town of Rehoboth in central Nambia, southern Africa (Wesuls et al. 2012). The nine quantitavie traits and abundances (percentage cover) of 87 species were measured in 378 plots. The traits include plant maximum height, above cover density (percentage cover of the plant canopy above a vertically projected contour of the plant), spine length, leaf ratio (leaf length divided by leaf width), leaf thickness, area of a leaf, specific leaf area, seed length, and diaspore length.

The Lieu-dit Aravo data set (Choler 2005) was collected in the southwestern Alps (Lieu-dit Aravo, Commune de Valloire, France). This data set includes the information on eight traits and abundances (percentage cover) of 82 alpine plant species in 75 sites. The traits are vegetative height, maximum lateral spread of clonal plants, leaf angle (leaf elevation angle estimated at the middle of the lamina), area of a leaf, leaf thickness (maximum thickness of a leaf cross section and avoiding the midrib), specific leaf area, leaf nitrogen concentration, and seed mass.

The Mount John data set is from a trial in the New Zealand short-tussock grasslands, located between the Mount John and Lake Alexandrina (Scott 1999). This trial is known as one of the longest running ecological experiments in New Zealand (>30 years). In 1982, a total of 30 plots of 8 × 50 m were established. We used seven quantitative traits measured on 51 plant species from this data set (Laliberté 2011).

The trait seed mass was not used, because missing values of this trait exist for 11 species. Two species were also deleted because of the incomplete measurement of traits. The first species coded as Cera_font is present in seven plots with its relative abundance ranging from 0.002% to 0.054%. The second species coded as Pter_veno is present only in one plot with a relative abundance 0.002%. Therefore, the deletion of these two species might have little effect on the results. The traits are reproductive height, leaf dry matter content, leaf nitrogen concentration, leaf phosphorous concentration, leaf sulfur concentration, specific leaf area, area of a leaf, and seed mass. Percentage cover was measured as relative abundance of each species.

References

Choler, P. Consistent shifts in alpine plant traits along a mesotopographical gradient. *Arct. Antarct. Alp. Res.* **37,** 444-453 (2005).

Heisse, K., Roscher, C., Schumacher, J. & Schulze, E.D. Establishment of grassland species in monocultures: different strategies lead to success. *Oecologia* **152,** 435-447 (2007).

Laliberté, E. *Land-use intensification in grazing systems: plant trait responses and feedbacks to ecosystem functioning and resilience*. PhD thesis, University of Canterbury, Christchurch (2011).

Pérez-Harguindeguy, N. *et al*. New handbook for standardised measurement of plant functional traits worldwide. *Australian Journal of Botany*, **61,** 167-234 (2013).

Roscher, C. *et al*. The role of biodiversity for element cycling and trophic interactions: an experimental approach in a grassland community. *Basic Appl. Ecol.* **5,** 107-121 (2004).

Scott, D. Sustainability of New Zealand high-country pastures under contrasting development inputs. 1. Site, and shoot nutrients. *New Zealand Journal of Agricultural Research*, **42,** 365-383 (1999).

Shipley, B., Laughlin, D.C., Sonnier, G. & Otfinowski, R. A strong test of a maximum entropy model of trait-based community assembly. *Ecology* **92,** 507-517 (2011).

Weigelt, A. et al. The Jena Experiment: six years of data from a grassland biodiversity experiment. *Ecology* **91,** 930 (2010).

Wesuls, D., Oldeland, J. & Dray, S. Disentangling plant trait responses to livestock grazing from spatio-temporal variation: the partial RLQ approach. *J. Veg. Sci.* **23,** 98-113 (2012).

Supplementary Table S1. The six data sets used

Data set	Quadrat number	Species number[1]	Trait number					Species richness
			Whole-plant	Leaf	Root	Regenerative	Total	(Range)
Loess Plateau, China	96	27	2	8	3		13	2 - 13
Arizona, USA	96	79	1	7	3	2	13	1 - 17
Jena, German	3281	58	5	3	1	2	11	1 - 28
Rehoboth, Namibia	378	87	3	4		2	9	1 - 28
Lieu-dit Aravo, France	75	82	2	5		1	8	5 - 29
Mount John, New Zealand	30	51	1	6			7	12 - 31

[1] Species number was the total of species present in all the quadrats.

Supplementary Table S2. The abbreviations for the traits. The mark √ indicates that the trait is included in the data set.

Traits	Abbreviations	Loess Plateau, China	Arizona, USA	Jena, German	Rehoboth, Namibia	Lieu-dit Aravo, France	Mount John, New Zealand
Whole-plant Traits							
Vegetative height	Hv	√	√			√	
Reproductive height	Hr						√
	height (not specified)				√		
Root mass fraction	RMF	√		√			
Dry mass per shoot	ShootMass			√			
Stem mass fraction	SMF			√			
Leaf mass fraction	LMF			√			
Leaf area ratio	LAR			√			
Above cover density	ACD				√		
Lateral spread of plants	Spread					√	
Spine length	SpiLen				√		
Leaf traits							
Area of a leaf	Area	√	√		√	√	√
Leaf dry matter content	LDMC	√	√				√
Specific leaf area	SLA	√	√	√	√	√	√
Mass-based leaf nitrogen concentration	LN	√		√		√	
Area-based leaf nitrogen concentration	LNa			√			
Leaf carbon concentration	LC	√	√				
Leaf phosphorous concentration	LP	√	√				√
Leaf sulfur concentration	LS						√

Supplementary Table S2. The abbreviations for the traits (continued).

Traits	Abbreviations	Loess Plateau, China	Arizona, USA	Jena, German	Rehoboth, Namibia	Lieu-dit Aravo, France	Mount John, New Zealand
Leaf traits							
Leaf $\delta^{13}C$	L13C		√				
Leaf $\delta^{15}N$	L15N		√				
Leaf ratio	LWRatio				√		
Leaf thickness	LT				√		
Leaf angle	LAngle					√	
Force to tear of leaves	LFt	√				√	
Force to punch of leaves	LFp	√					
Below-ground traits							
Depth of roots	RDepth	√		√			
Lateral extent of roots	RLateral	√					
Specific root length	SRL		√				
Fine-root carbon concentration	RC		√				
Fine-root nitrogen concentration	RN		√				
Force to tear of roots	RFt	√					
Regenerative traits							
Seed mass	SeedMass		√	√		√	√
Seed length	SeedLen				√		
Diaspore length	DiaLen				√		
Time to seedling emergence	SeedEmerg			√			
Flowering duration	FlrDuration		√				

Supplementary Table S3. Trait correlation in the Loess Plateau data set. The upper/right part shows correlation coefficients calculated pairwisely, and the lower/left part contains the corresponding P values. The P values smaller than 0.05 are in bold. About 26% (20 coefficients) of 78 correlation coefficients are significant ($P \leq 0.05$). See the abbreviations for traits in the Table S2.

	Hv	Area	LDMC	SLA	LC	LN	LP	LFt	LFp	RFt	RDepth	RLateral	RMF
Hv		0.04	0.39	0.02	0.55	0.21	0.04	0.30	0.03	-0.18	0.37	0.29	-0.22
Area	0.85		-0.05	0.11	-0.25	-0.20	-0.02	-0.03	0.16	0.34	0.47	0.09	0.10
LDMC	**0.05**	0.79		-0.48	0.56	-0.39	-0.67	0.58	0.49	-0.06	0.14	0.34	0.27
SLA	0.91	0.57	**0.01**		-0.04	0.19	0.40	-0.17	-0.23	0.11	-0.15	-0.24	-0.40
LC	**0.00**	0.20	**0.00**	0.85		0.00	-0.14	0.22	0.02	-0.07	0.24	0.51	0.05
LN	0.29	0.33	**0.04**	0.34	0.99		0.71	-0.38	-0.43	0.00	0.26	-0.11	-0.24
LP	0.86	0.91	**0.00**	**0.04**	0.50	**0.00**		-0.54	-0.48	0.08	0.22	-0.02	-0.15
LFt	0.12	0.89	**0.00**	0.39	0.27	**0.05**	**0.00**		0.41	-0.01	-0.07	0.05	-0.03
LFp	0.89	0.42	**0.01**	0.26	0.94	**0.02**	**0.01**	**0.03**		0.20	-0.16	-0.20	0.08
RFt	0.37	0.08	0.75	0.59	0.72	1.00	0.70	0.97	0.31		-0.07	-0.10	-0.12
RDepth	0.06	**0.01**	0.48	0.44	0.22	0.18	0.26	0.71	0.41	0.72		0.66	0.18
RLateral	0.14	0.64	0.08	0.22	**0.01**	0.59	0.93	0.81	0.31	0.61	**0.00**		0.43
RMF	0.27	0.61	0.18	**0.04**	0.79	0.23	0.46	0.87	0.68	0.57	0.36	**0.02**	

Supplementary Table S4. Trait correlation in the Arizona data set. The upper/right part shows correlation coefficients calculated pairwisely, and the lower/left part contains the corresponding P values. The P values smaller than 0.05 are in bold. About 32% (25 coefficients) of 78 correlation coefficients are significant ($P \leq 0.05$). See the abbreviations for traits in the Table S2.

	Hv	Area	LDMC	SLA	SRL	SeedMass	FlrDuration	LC	LN	L13C	L15N	RC	RN
Hv		0.51	0.22	-0.14	-0.15	0.29	-0.14	0.11	-0.06	-0.03	0.01	0.01	-0.05
Area	**0.00**		-0.01	-0.04	-0.22	0.18	0.22	0.17	0.09	0.13	-0.10	0.00	0.00
LDMC	**0.06**	0.91		-0.47	-0.20	-0.09	0.03	0.24	-0.50	-0.34	-0.15	0.08	-0.33
SLA	0.22	0.71	**0.00**		0.44	-0.04	-0.08	-0.25	0.51	0.02	0.04	-0.14	0.29
SRL	0.20	**0.05**	0.08	**0.00**		-0.30	-0.36	-0.34	0.07	-0.47	0.23	-0.01	0.02
SeedMass	**0.01**	0.12	0.45	0.74	**0.01**		0.16	0.09	0.41	0.17	0.00	0.01	0.39
FlrDuration	0.20	**0.05**	0.78	0.50	**0.00**	0.15		0.01	0.16	0.14	-0.01	0.02	0.20
LC	0.34	0.14	**0.03**	**0.02**	**0.00**	0.45	0.95		-0.14	0.12	-0.30	0.09	-0.14
LN	0.60	0.43	**0.00**	**0.00**	0.53	**0.00**	0.16	0.21		0.24	0.13	-0.13	0.64
L13C	0.78	0.27	**0.00**	0.88	**0.00**	0.12	0.22	0.28	**0.03**		-0.20	-0.10	-0.01
L15N	0.92	0.40	0.19	0.74	**0.04**	0.97	0.95	**0.01**	0.25	0.08		0.12	0.26
RC	0.96	0.99	0.49	0.21	0.96	0.92	0.86	0.41	0.25	0.38	0.31		-0.04
RN	0.64	0.99	**0.00**	**0.01**	0.85	**0.00**	0.08	0.22	**0.00**	0.93	**0.02**	0.70	

Supplementary Table S5. Trait correlation in the Jena data set. The upper/right part shows correlation coefficients calculated pairwisely, and the lower/left part contains the corresponding P values. The P values smaller than 0.05 are in bold. About 58% (32 coefficients) of 55 correlation coefficients are significant ($P \leq 0.05$). See the abbreviations for traits in the Table S2.

	SeedMass	SeedEmerg	RDepth	RMF	ShootMass	SMF	LMF	SLA	LAR	LN	LNa
SeedMass		-0.23	0.39	-0.14	0.07	0.21	-0.16	-0.13	-0.19	0.45	0.51
SeedEmerg	0.09		-0.43	0.29	-0.25	-0.30	0.31	-0.15	0.18	0.03	0.29
RDepth	**0.00**	**0.00**		-0.20	0.54	0.31	-0.31	0.32	-0.05	0.42	0.07
RMF	0.28	**0.03**	0.14		-0.35	-0.53	0.54	-0.32	0.31	-0.25	0.00
ShootMass	0.61	0.06	**0.00**	**0.01**		0.22	-0.28	0.31	-0.06	0.21	-0.04
SMF	0.11	**0.02**	**0.02**	**0.00**	0.10		-0.98	0.49	-0.68	0.46	-0.03
LMF	0.23	**0.02**	**0.02**	**0.00**	**0.03**	**0.00**		-0.49	0.71	-0.44	0.05
SLA	0.33	0.28	**0.02**	**0.01**	**0.02**	**0.00**	**0.00**		0.22	0.35	-0.48
LAR	0.15	0.18	0.72	**0.02**	0.67	**0.00**	**0.00**	0.10		-0.26	-0.35
LN	**0.00**	0.84	**0.00**	0.06	0.12	**0.00**	**0.00**	**0.01**	**0.05**		0.55
LNa	**0.00**	**0.03**	0.59	0.99	0.79	0.80	0.72	**0.00**	**0.01**	**0.00**	

Supplementary Table S6. Trait correlation in the Rehoboth data set. The upper/right part shows correlation coefficients calculated pairwisely, and the lower/left part contains the corresponding P values. The P values smaller than 0.05 are in bold. About 39% (14 coefficients) of 36 correlation coefficients are significant (P ≤ 0.05). See the abbreviations for traits in the Table S2.

	height	ACD	LWRatio	LT	Area	SLA	SpineLen	SeedLen	DiaLen
height		-0.04	-0.04	0.11	-0.01	-0.21	0.54	0.52	0.10
ACD	0.74		-0.23	0.19	0.12	-0.22	0.03	0.16	0.14
LWRatio	0.69	**0.03**		-0.21	-0.06	-0.15	-0.13	-0.01	0.01
LT	0.33	0.08	**0.05**		0.32	-0.09	0.16	0.33	0.41
Area	0.92	0.26	0.56	**0.00**		0.03	-0.04	0.26	0.10
SLA	**0.05**	**0.04**	0.16	0.43	0.79		-0.23	-0.22	-0.03
SpineLen	**0.00**	0.75	0.25	0.13	0.70	**0.03**		0.45	0.06
SeedLen	**0.00**	0.15	0.96	**0.00**	**0.02**	**0.04**	**0.00**		0.34
DiaLen	0.34	0.18	0.91	**0.00**	0.35	0.77	0.58	**0.00**	

Supplementary Table S7. Trait correlation in the Lieu-dit Aravo data set. The upper/right part shows correlation coefficients calculated pairwisely, and the lower/left part contains the corresponding P values. The P values smaller than 0.05 are in bold. About 29% (8 coefficients) of 28 correlation coefficients are significant (P ≤ 0.05). See the abbreviations for traits in the Table S2.

	Hv	Spread	LAngle	Area	LT	SLA	LN	SeedMass
Hv		-0.22	0.41	0.12	-0.07	-0.14	-0.11	0.15
Spread	**0.05**		-0.17	0.09	-0.19	-0.04	0.14	0.03
LAngle	**0.00**	0.12		-0.33	0.17	-0.33	-0.20	0.07
Area	0.29	0.41	**0.00**		-0.07	0.20	0.06	0.26
LT	0.55	0.09	0.13	0.53		-0.39	-0.41	0.02
SLA	0.22	0.69	**0.00**	0.07	**0.00**		0.75	-0.09
LN	0.34	0.21	0.07	0.62	**0.00**	**0.00**		0.10
SeedMass	0.17	0.80	0.53	**0.02**	0.84	0.44	0.35	

Supplementary Table S8. Trait correlation in the Mount John data set. The upper/right part shows correlation coefficients calculated pairwisely, and the lower/left part contains the corresponding P values. The P values smaller than 0.05 are in bold. About 62% (13 coefficients) of 21 correlation coefficients are significant (P ≤ 0.05). See the abbreviations for traits in the Table S2.

	Hr	LDMC	LN	LP	LS	SLA	Area
Hr		-0.01	0.24	0.02	0.11	0.19	0.50
LDMC	0.95		-0.68	-0.67	-0.54	-0.64	-0.33
LN	0.08	**0.00**		0.61	0.75	0.64	0.37
LP	0.89	**0.00**	**0.00**		0.77	0.62	0.15
LS	0.44	**0.00**	**0.00**	**0.00**		0.60	0.12
SLA	0.19	**0.00**	**0.00**	**0.00**	**0.00**		0.05
Area	**0.00**	**0.02**	**0.01**	0.31	0.40	0.71	

134 **Supplementary Table S9. Estimations of the intrinsic dimensionality of traits for the six data sets.**

Method	Loess Plateau, China	Arizona, USA	Jena, German	Rehoboth, Namibia	Lieu-dit Aravo, France	Mount John, New Zealand
Cattell's scree test (Cattell 1966)	4	4	4	3	4	2
Kaiser's rule (Kaiser 1960)	4	4	4	4	4	2
Parallel analysis (Horn 1965)	4	4	3	2	3	2

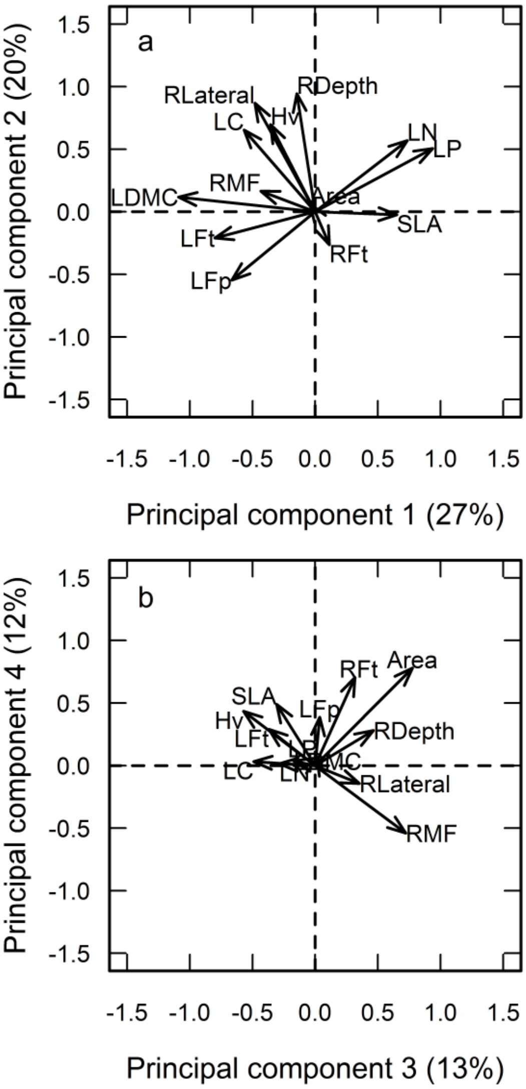

Supplementary Figure S1. Principal component analysis for the traits in the Loess Plateau data set. Leaf dry matter content and depth of roots had the highest loadings in the first two axes, respectively. Area of a leaf had the highest loadings in the third and fourth axes. These three traits were used to calculate functional diversity indices in the HL method. See the abbreviations for traits in the Table S2. Values in parentheses after the titles of the coordinates indicate the percentage of the total variance accounted for by each axis.

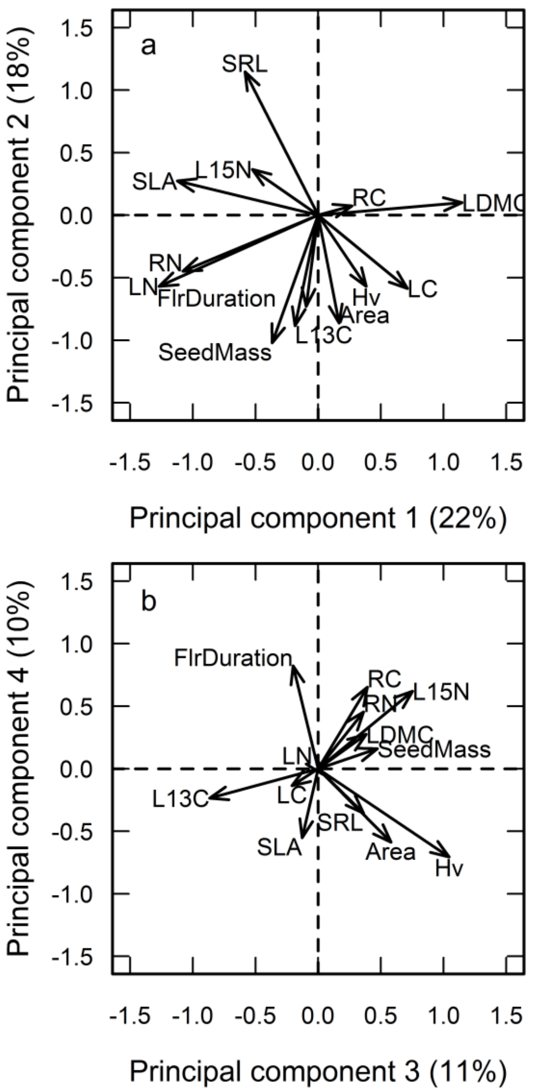

Supplementary Figure S2. Principal component analysis for the traits in the Arizona data set. Leaf nitrogen concentration, specific root length, vegetative height, and flowering duration had the highest loadings in the first four axes, respectively. These four traits were used to calculate functional diversity indices in the HL method. See the abbreviations for traits in the Table S2. Values in parentheses after the titles of the coordinates indicate the percentage of the total variance accounted for by each axis.

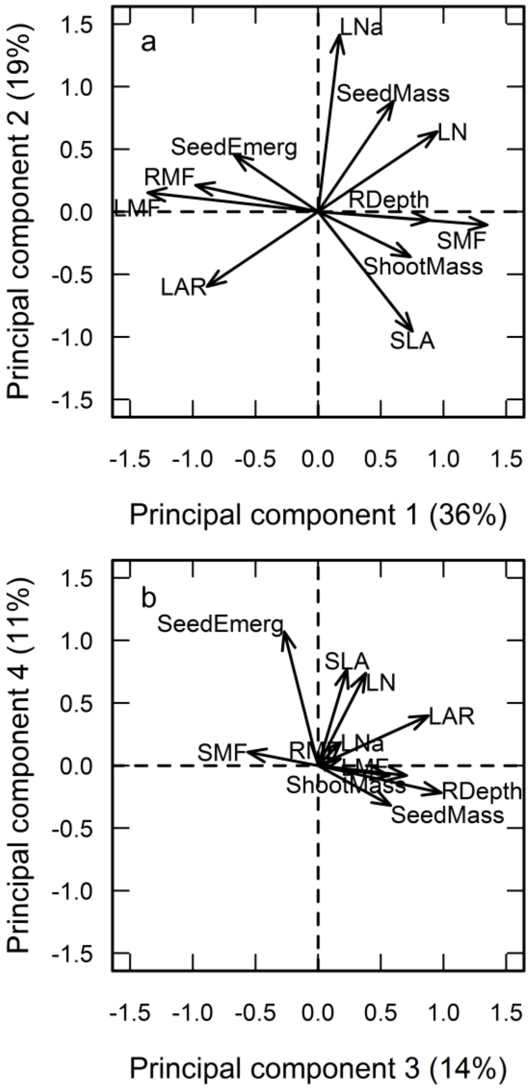

Supplementary Figure S3. Principal component analysis for the traits in the Jena data set. Leaf mass fraction, area based leaf nitrogen concentration, depth of roots, and time to seedling emergence had the highest loadings in the first four axes, respectively. These four traits were used to calculate functional diversity indices in the HL method. See the abbreviations for traits in the Table S2. Values in parentheses after the titles of the coordinates indicate the percentage of the total variance accounted for by each axis.

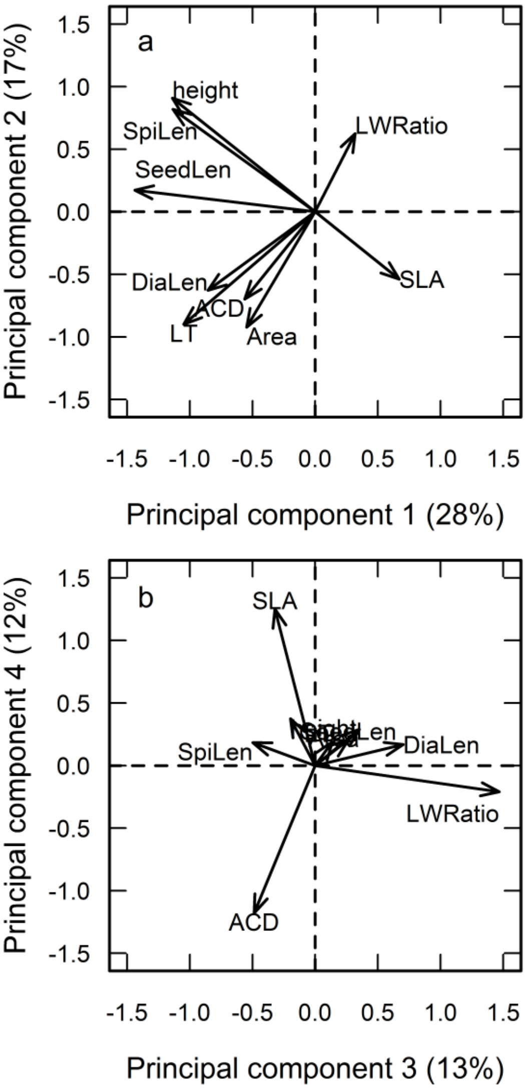

Supplementary Figure S4. Principal component analysis for the traits in the Rehoboth data set. Seed length, area of a leaf, leaf ratio, and specific leaf area had the highest loadings in the first four axes, respectively. These four traits were used to calculate functional diversity indices in the HL method. See the abbreviations for traits in the Table S2. Values in parentheses after the titles of the coordinates indicate the percentage of the total variance accounted for by each axis.

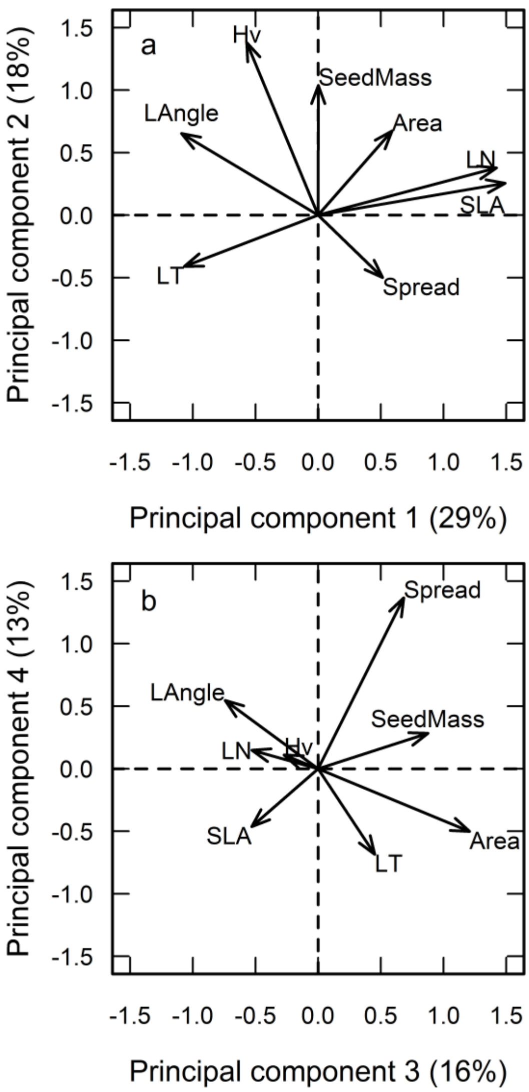

Supplementary Figure S5. Principal component analysis for the traits in the Lieu-dit Aravo data set. Specific leaf area, vegetative height, area of a leaf, and maximum lateral spread of clonal plants had the highest loadings in the first four axes, respectively. These four traits were used to calculate functional diversity indices in the HL method. See the abbreviations for traits in the Table S2. Values in parentheses after the titles of the coordinates indicate the percentage of the total variance accounted for by each axis.

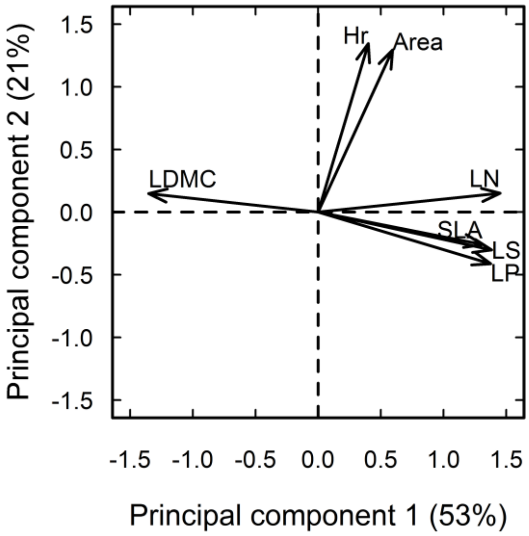

Supplementary Figure S6. Principal component analysis for the traits in the Mount John data set. Leaf nitrogen concentration and reproductive height had the highest loadings in the first two axes, respectively. These two traits were used to calculate functional diversity indices in the HL method. See the abbreviations for traits in the Table S2. Values in parentheses after the titles of the coordinates indicate the percentage of the total variance accounted for by each axis.
